# Non‐Coordinated Phenolate Anions and Their Application in SF_6_ Activation

**DOI:** 10.1002/chem.202003504

**Published:** 2020-11-03

**Authors:** Robin F. Weitkamp, Beate Neumann, Hans‐Georg Stammler, Berthold Hoge

**Affiliations:** ^1^ Centrum für Molekulare Materialien Fakultät für Chemie Universität Bielefeld Universitätsstraße 25 33615 Bielefeld Germany

**Keywords:** hydrogen bond, phenol, phosphazene base, SF_6_ activation, weakly coordinating cation

## Abstract

The reaction of the strong monophosphazene base with the weakly acidic phenol leads to the formation of a phenol–phenolate anion with a moderately strong hydrogen bond. Application of the more powerful tetraphosphazene base (Schwesinger base) renders the isolation of the corresponding salt with a free phenolate anion possible. This compound represents the first species featuring the free phenolate anion [H_5_C_6_‐O]^−^. The deprotonation of phenol derivatives with tetraphosphazene bases represents a great way for the clean preparation of salts featuring free phenolate anions and in addition allows the selective syntheses of hydrogen bonded phenol‐phenolate salts. This work presents a phosphazenium phenolate salt with a redox potential of −0.72 V and its capability for the selective activation of the chemically inert greenhouse gas SF_6_. The performed two‐electron reduction of SF_6_ leads to phosphazenium pentafluorosulfanide ([SF_5_]^−^) and fluoride salts.

## Graphical abstract

**Non‐coordinated phenolate anions**: By deprotonation of phenol with the strong tetraphosphazene Schwesinger base a salt of the so far unknown free phenolate anion was formed. In contrast to that, the deprotonation with a less basic monophosphazene base leads to a phenol–phenolate salt with an additional hydrogen bond to the phosphazenium cation. Applying electron‐rich phenolate salts we further present the two‐electron reduction of SF_6_ leading to [SF_5_]^−^ and F^−^ salts.

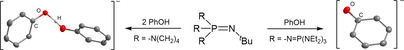

Phenol represents the simplest aromatic alcohol, and thus has been in the focus of numerous theoretical calculations[^1^, ^2^, ^3^, ^4^] as well as practical applications.[^5^, ^6^] Especially sodium phenolate has emerged as a highly important bulk chemical for the industrial production of salicylic acid in the Kolbe–Schmitt process.[^7^, ^8^]

Fundamental reactions in the biosphere are strongly dependent on phenolic species. The amino acid tyrosine (*p*‐hydroxyphenylalanine) is crucial for the success of photosynthesis, as tyrosine is photo‐oxidized in the oxygen evolving complex (OEC) of the photosystem II via a proton coupled electron transfer (PCET) reaction with a hydrogen bonded histidine.^[9]^ Hydrogen bonds of phenol are strongly governing the acidity of OH functions, which turned out to be crucial in several biological and chemical systems.[^3^, ^4^, ^5^, ^10^, ^11^]

With regard to the great importance of phenol, it is surprising that the molecular structure and the characteristics of the non‐coordinated phenolate anion have not been unambiguously documented.

Phenol with a p*K*_BH_^+^ value of 9.98[^1^, ^12^] is weakly acidic and is easily deprotonated by alkali hydroxides or hydrides to yield the corresponding metal phenolates.[^5^, ^7^, ^13^]

Fraser et al. reported on the separation of sodium and potassium cations from phenolates^[15]^ and phenol‐phenolate salts^[16]^ by means of crown ethers. For the latter they reported short hydrogen bonds with O−O distances of 247.1(3) pm to 248(1) pm. The strong tendency of hydrogen bonding is also observed in the imidazolium salt of Clyburne and co‐workers (Figure 1, right), which exhibits strong cation‐anion interactions.^[14]^

**Figure 1:**
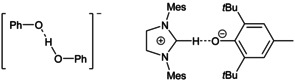
Structures of the phenol‐phenolate anion^[11]^ and an NHC adduct of a phenol derivative.^[14]^

Reetz et al. used tetra‐*n‐*butylammonium hydroxide for the deprotonation of phenol to generate a free [H_5_C_6_‐O]^−^ anion without cation‐anion interactions. All attempts to isolate the phenolate anion were thwarted by the selective formation of the phenol‐phenolate adduct (Figure 1, left).^[11]^ Davidson applied phosphonium ylides for the deprotonation of phenols resulting in salts featuring short cation‐anion C−H⋅⋅⋅O^−^ hydrogen bonds.[^17^, ^18^] In addition to that, numerous substituted phenol derivatives containing electron‐withdrawing groups, thus featuring an increased acidity, were investigated.[^6^, ^19^]

However, no example of the non‐coordinated phenolate anion [H_5_C_6_‐O]^−^ was reported so far. The structural characteristics of mono‐ and tetraphosphazene bases like **1** and **2**,^[20]^ presented in Scheme 1 and Scheme 2, seem promising for the design of systems featuring the free phenolate anion, as well as phenolate derivatives containing electron‐donating groups.

**Scheme 1:**
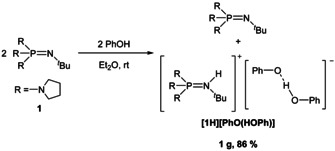
Synthesis of **[1H][PhO(HOPh)]**.

**Scheme 2:**
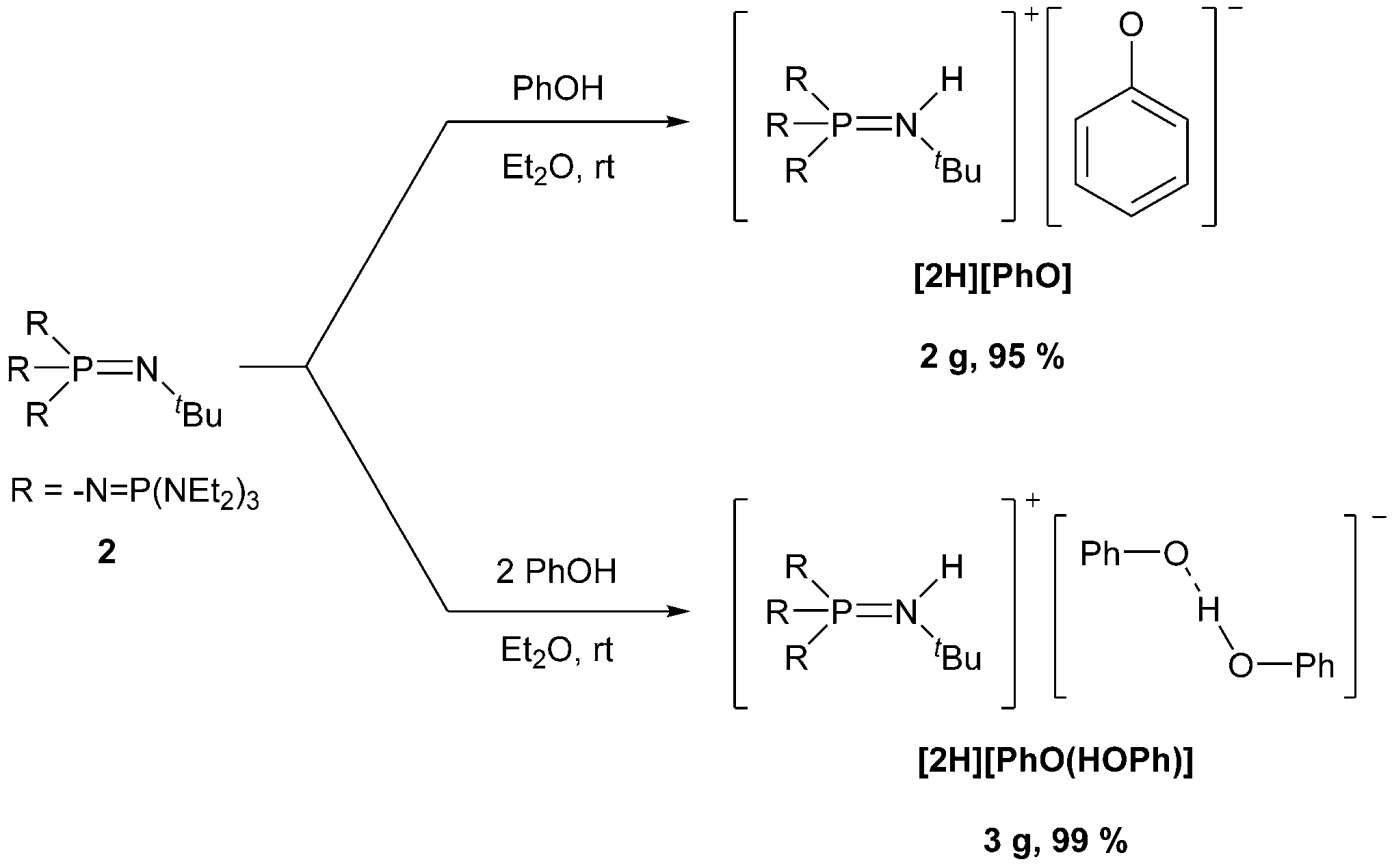
Synthesis of phenolate salts using phosphazene **2**.

The deprotonation of phenol with equimolar quantities of the commercially available pyrrolidino phosphazene **1** in diethyl ether leads to the precipitation of a light brown oil.^[21]^ The ^31^P NMR spectrum of the supernatant shows the signal of the free base at *δ*=−10.3 ppm. Thus, the basicity of the pyrrolidino phosphazene **1** is not sufficient for the complete deprotonation of phenol and solely affords a phenol‐phenolate adduct (Scheme 1, Figure 2).^[22]^

**Figure 2:**
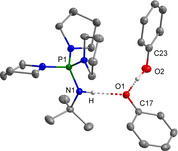
Molecular structure of **[1H][PhO(HOPh)]**.^[23]^ Selected bond lengths [pm] and angles [°]: O1−O2 249.1(1), N1−O1 279.2(1), O1−C17 131.9(2); N1‐O1‐O2 112.8(1).

Salt **[1H][PhO(HOPh)]** crystallizes from the reaction mixture at −28 °C in an 84 % yield.^[21]^ In the ^31^P NMR spectrum of the product a signal at *δ*=22.2 ppm is observed, which is due to the protonated phosphazene **[1H]^+^**.

With regard to familiar O−O distances in [OH(OH_2_)]^−^ (229 pm),^[24]^ [H_3_O(H_2_O)]^+^ (249 pm)^[25]^ and water aggregates (283 pm),^[25]^ the phenolate anion exhibits moderately strong hydrogen bonding to the phenol molecule with an O1−O2 distance of 249.1(2) pm, which is well comparable with the literature data.[^11^, ^16^] Furthermore, an additional interaction of the phenolate anion with the iminium proton (N1−O1 279.2(1) pm) is observed.

This clearly requires more basic and sterically encumbered phosphazenes like **2** for the separation of non‐coordinated phenolates (Scheme 2).

The reaction of equimolar quantities of phenol and **2** leads to the precipitation of the expected phenolate **[2H][PhO]** (Figure 3) as colorless crystals in yields up to 95 %.^[21]^ The product is highly air sensitive and decomposes above 75 °C. The decomposition of the product in [D_1_]chloroform and [D_3_]acetonitrile solution was observed by ^13^C NMR spectroscopy and led to deep blue and strong yellow solutions, respectively, whose color eventually faded.^[21]^

**Figure 3:**
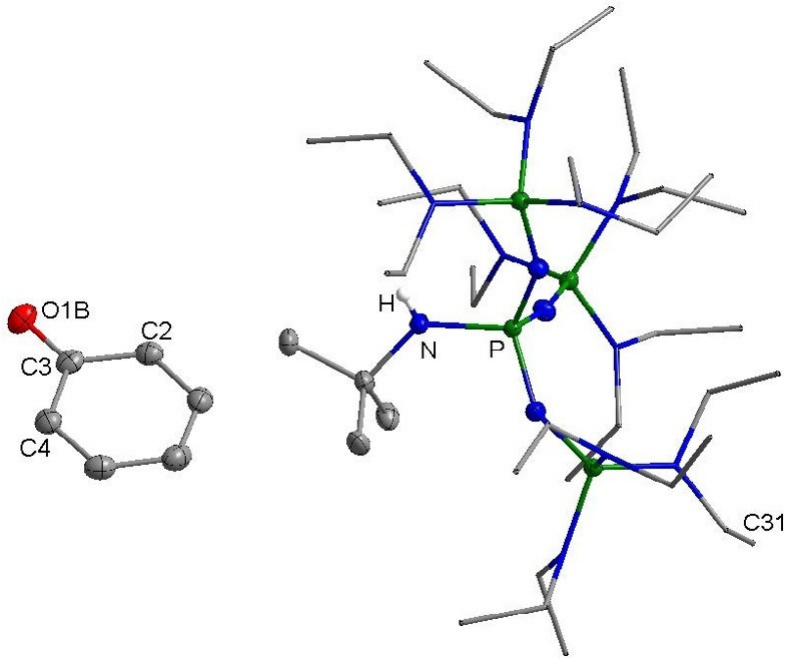
Molecular structure of the salt **[2H][PhO]**.^[23]^ The anion is disordered (94:6). Selected bond lengths [pm]: O1B−C3 128.7(2), C2−C3 143.6(2), C3−C4 142.8(2).

Salt **[2H][PhO]** is the first example of the non‐coordinated phenolate anion. The anion is disordered in a ratio of 94:6.^[22]^ In the major representative the closest C−H⋅⋅⋅O^−^ contact of cation and anion (O1B−C31) was determined to 325.9(2) pm, which is in the range of C−H⋅⋅⋅O^−^ hydrogen bonds.[^18^, ^26^] The C−O bond length of the anion in **[2H][PhO]** amounts to 128.7(2) pm and is thus significantly shortened in comparison to the C−O bonds of coordinated anions as present in sodium phenolate (133(1) pm)^[27]^ or in **[1H][PhO(HOPh)]** (131.9(2) pm). This bond shortage points to a significant resonance stabilization of the negative charge, which is also confirmed by a strong upfield shift (*δ*=5.5 ppm) of the signal of the *para* positioned proton in the ^1^H NMR spectrum (Figure 4, top). The C−C distances in free [PhO]^−^ are slight elongated (138.6(1) pm to 143.6(1) pm) compared to sodium phenolate (138(1) pm to 142(1) pm). The corresponding angles within the aromatic system do not differ significantly.

**Figure 4:**
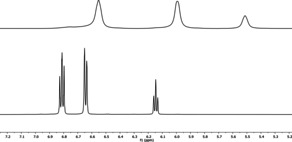
Aromatic region of the ^1^H NMR spectra of **[2H][PhO]** (top) and **[2H][PhO(HOPh)]** (bottom) in [D_8_]THF.

Application of two equivalents of phenol allows the synthesis of the phenol‐phenolate compound **[2H][PhO(HOPh)]** (Figure 5) in excellent yields (99 %, Scheme 2). The phenol‐phenolate salt exhibits a higher thermal stability (m.p. 125 °C) and deteriorates less eagerly in air or in [D_1_]chloroform and [D_3_]acetonitrile solution than the corresponding non‐coordinated phenolate salt **[2H][PhO]**.^[21]^ Hydrogen bonding brings about downfield shifts of the aromatic protons in the ^1^H NMR spectrum and clean couplings (Figure 4, bottom).

**Figure 5:**
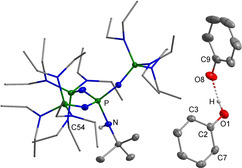
Molecular structure of the salt **[2H][PhO(HOPh)]**.^[23]^ The donor hydrogen atom is disordered at both oxygen atoms with a ratio of 1:1, only one is shown. Selected bond lengths [pm]: O1−O8 243.7(2), O1−C2 131.9(2), O8−C9 132.1(2).

The associated hydrogen bond with an O1−O8 distance of 243.7(2) pm is shortened in comparison to **[1H][PhO(HOPh)]** (249.1(1) pm).^[22]^

Several papers addressed the redox potentials of various phenols^[28]^ and phenolates[^29^, ^30^] as determined by (cyclic) voltammetry, preferentially in acetonitrile solution. The anions were preferentially generated in situ via deprotonation with tetraalkylammonium hydroxides. The unsuccessful preparation of the free phenolate anion by deprotonation with ammonium hydroxides^[11]^ and the fast deterioration of non‐coordinated phenolates like **[2H][PhO]** in acetonitrile^[21]^ casts doubt on the reported redox potentials.

The now possible selective synthesis of hydrogen bonded phenolate moieties makes the disclosure of the influence of hydrogen bonding on the redox properties of phenolate anions via cyclic voltammetry (CV) conceivable (Figure 6). The rapid reactions of intermediates led to irreversible oxidation processes at low scan rates of 100 mV s^−1^. Thus, only oxidation potentials (*E*_Ox_) can be determined, which are compared with quantum chemical calculations on the BP86/6–311+g(3df,2p) level.^[31]^

**Figure 6:**
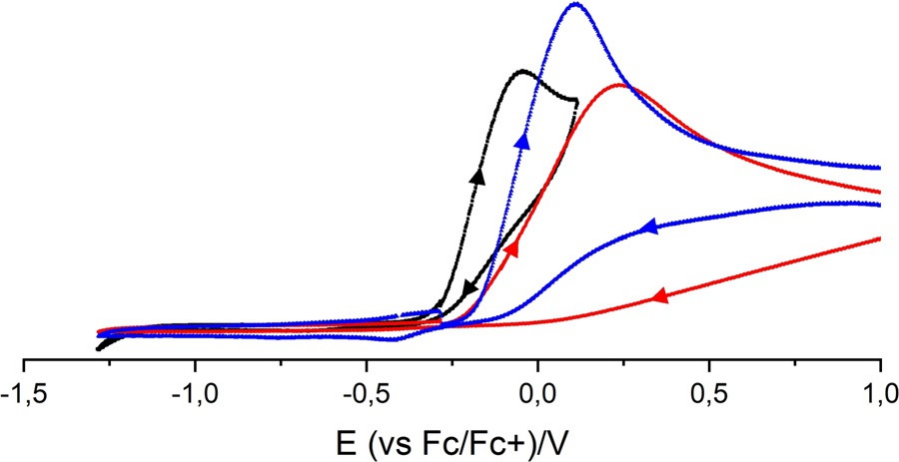
Cyclic voltammograms of **[2H][PhO]** (black), **[2H][PhO(HOPh)]** (red) and **[2H][PhO(HOPh)]** + excess H_2_O (concentration of 0.1 m H_2_O in the electrolyte solution, blue), recorded in 0.1 m [NBu_4_][PF_6_]⋅THF solution at 100 mV s^−1^.^[21]^ Fc/Fc^+^ was set at +0.405 V.

Salt **[2H][PhO]** was oxidized in THF solution at *E*_Ox_=−0.12(1) V vs. the Fc/Fc^+^ couple (black, Figure 6).^[21]^ This value is cathodically shifted in comparison to the estimated value in acetonitrile solution reported in the literature (+0.24 V).^[30]^ Interestingly, the hydrogen bonded phenol‐phenolate adduct is oxidized at a more positive potential (*E*_Ox_=+0.22(1) V), and resembles the potential reported for the phenolate/phenoxyl couple (+0.24 V).^[30]^ The anodically shifted oxidation potential of [PhO(HOPh)]^−^ is rationalized by a reduced charge density of the phenolate oxygen in comparison to free [PhO]^−^. A concentration of 0.1 m H_2_O (17 equivalents) was prepared by adding water to the phenol‐phenolate electrolyte solution, which leads to a cathodic shift of *E*_Ox_ (+0.10(1) V, Figure 6). Likewise the addition of water (0.1 m, 0.2 m, 0.7 m) to **[2H][PhO]** results in increasing potentials of *E*_Ox_=+0.02(1) V, +0.04(1) V and +0.11(1) V.^[21]^ This clearly underlines that hydrogen bonded adducts of phenolates instead of free phenolates have been oxidized previously.

The presented tendency is confirmed by the calculation of adiabatic ionization potentials (*E*_i_) of phenolates in the gas phase.^[31]^ The influence of hydrogen bonding on the potential of the phenolate anion is more pronounced in the phenol adduct [PhO(HOPh)]^−^ (*E*_i_=314.90(1) kJ mol^−1^) than in the water adduct [PhO(H_2_O)]^−^ (*E*_i_=267.42(1) kJ mol^−1^), both significantly differ from the calculated value of the free anion [PhO]^−^ (*E*_i_=228.69(1) kJ mol^−1^).

For the employment of phenolates as strong reducing agents, we selected 2,6‐di‐*tert*‐butyl‐4‐methoxyphenol (^MeO*t*Bu2^PhOH) as the substrate of choice (Scheme 3).

**Scheme 3:**
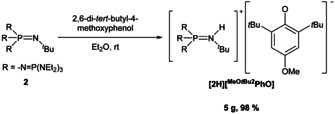
Synthesis of **[2H][^MeO*t*Bu2^PhO]**.

Deprotonation of this phenol with **2** clearly furnished the corresponding phenolate salt **[2H][^MeO*t*Bu2^PhO]** (Figure 7).^[21]^ The salt is significantly more air sensitive than the previously discussed phenolate. Air contact effects a quick color change from yellow to red‐brown.

**Figure 7:**
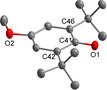
Molecular structure of the anion of **[2H][^MeO*t*Bu2^PhO]**.^[23]^ Selected bond lengths [pm]: C41−O1 129.0(2), C41−C42 144.8(4), C41−C46 144.9(4).

As the closest cation‐anion contact in **[2H][^MeO*t*Bu2^PhO]** a O1−C8 separation of 303.9(1) pm was observed.^[22]^ The O1−C41 bond (129.0(2) pm) is similar to that in the anion of **[2H][PhO]**.

The anion in **[2H][^MeO*t*Bu2^PhO]** undergoes a reversible redox reaction at *E* ^0^=−0.72(1) V vs. Fc/Fc^+^ (Figure 8), which is significantly lower than the literature data in acetonitrile (−0.45(1) V).[^29^, ^30^] Thus it has a similar redox potential as zinc and can be classified as an organic zinc reagent.^[32]^

**Figure 8:**
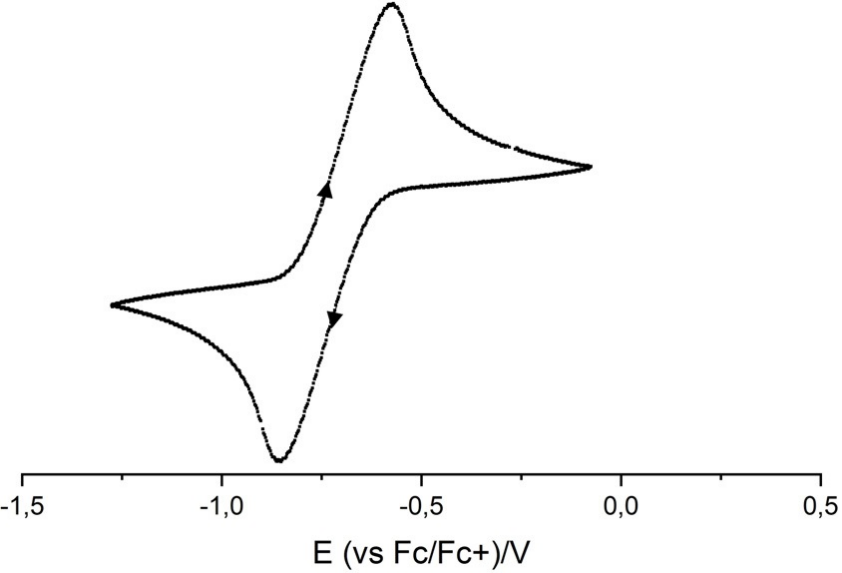
Cyclic voltammogram of **[2H][^MeO*t*Bu2^PhO]** recorded in 0.1 m [NBu_4_][PF_6_]⋅THF solution at 100 mV s^−1^.^[21]^ Fc/Fc^+^ was set at +0.405 V.

In order to demonstrate the reducing capability of **[2H][^MeO*t*Bu2^PhO]**, the reaction with the chemically inert sulfur hexafluoride was investigated (Scheme 4).

**Scheme 4:**
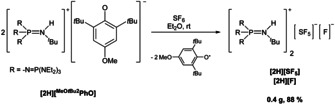
Activation of SF_6_ with phenolate **[2H][^MeO*t*Bu2^PhO]**.

SF_6_ is the most potent greenhouse gas known to date^[33]^ and has a dramatic impact on the climate due to its high chemical stability.^[34]^ Therefore the chemical degradation of SF_6_ has become an important issue of current research.[^35^, ^36^, ^37^, ^38^] In ethereal solution the treatment of the phenolate with SF_6_ (Scheme 4) was accompanied by a color change from yellow to pink to deep red. The formation of the [SF_5_]^−^ anion was evidenced by ^19^F NMR spectroscopy featuring a quintet at *δ*=88.7 ppm and a doublet at 59.5 ppm, with a coupling constant of ^2^ *J*_FF_=45 Hz (Figure 9).[^36^, ^39^]

**Figure 9:**
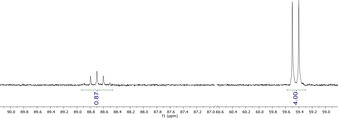
Resonances of the [SF_5_]^−^ anion in the ^19^F NMR spectrum of the reaction of **[2H][^MeO*t*Bu2^PhO]** with SF_6_.

The broad resonance of the fluoride anion in the product was observed in the ^19^F NMR spectrum at *δ*=−173.0 ppm.^[21]^ According to the favorable decomposition pathway,[^37^, ^38^] the formed [SF_6_]^⋅−^ radical anion disintegrates into a fluoride anion and an (SF_5_)**^.^** radical. The latter is further reduced by a second phenolate to obtain the [SF_5_]^−^ anion. The thermally stable salt mixture of **[2H][SF_5_]** and **[2H][F]** (dec.>123 °C) precipitates from the reaction mixture as a colorless solid in high yields (>88 %).^[21]^ The X‐ray structural analysis of a single crystal of **[2H][SF_5_]** obtained by slow precipitation from the reaction mixture confirms the presence of the [SF_5_]^−^ anion with its distorted pseudo square‐pyramidal geometry.[^21^, ^22^, ^36^, ^40^]

In conclusion we succeeded in the clean deprotonation of phenol and 2,6‐di‐*tert*‐butyl‐4‐methoxyphenol by means of the tetraphosphazene base **2**, affording salts of the free phenolate anions in **[2H][PhO]** and in **[2H][^MeO*t*Bu2^PhO]** in excellent yields (>95 %). The strength of the base as well as the stoichiometry determines if a phenol‐free phenolate salt or a phenol‐phenolate adduct is generated. The latter anions were preferentially obtained by deprotonation of phenol with the less basic pyrrolidino monophosphazene **1** or alternatively in the case of **[2H][PhO(HOPh)]** by the employment of two molar equivalents of phenol.

We also disclosed the successful degradation of sulfur hexafluoride (SF_6_) in a two‐electron reduction process applying **[2H][^MeO*t*Bu2^PhO]**, leading to the corresponding phosphazenium pentafluorosulfanide and fluoride salts **[2H][SF_5_]** and **[2H][F]** in high yields (>88 %). The use of phosphazenium phenolates for the preparation of highly reactive anions, especially radical anions, is under active study in our laboratory.

## Experimental Section

**Crystallographic data**: Deposition numbers 1973242, 1973243, 1973244, 2002668, and 2002669 contain the supplementary crystallographic data for this paper. These data are provided free of charge by the joint Cambridge Crystallographic Data Centre and Fachinformationszentrum Karlsruhe Access Structures service.

## Conflict of interest

The authors declare no conflict of interest.

## Supporting information

As a service to our authors and readers, this journal provides supporting information supplied by the authors. Such materials are peer reviewed and may be re‐organized for online delivery, but are not copy‐edited or typeset. Technical support issues arising from supporting information (other than missing files) should be addressed to the authors.

Supplementary
