# Supplementary material for: Non‐Coordinated Phenolate Anions and Their Application in SF6 Activation
Source: Chemistry. 2020 Nov 3;27(21):6460–4. doi: 10.1002/chem.202003504 (PMC8247349; doi:10.1002/chem.202003504)
Supplement: Supplementary file 1 — Supplementary [file CHEM-27-6460-s001.pdf]

Chemistry–A European Journal

Supporting Information

Non-Coordinated Phenolate Anions and Their Application in SF_6 Activation

Robin F. Weitkamp, Beate Neumann, Hans-Georg Stammler, and Berthold Hoge*[a]

1. Experimental Section

1.1 General Part

All chemicals were obtained from commercial sources and used without further purification. Standard high-vacuum techniques were employed throughout all experiments. Non-volatile compounds were handled in a dry N_2 atmosphere using Schlenk techniques.

1.2 Analysis Methods

1.2.1 NMR Spectroscopy

NMR spectra were recorded on a Bruker Avance III 500 spectrometer (^{1}H 500.01 MHz; ^{13}C 125.73 MHz; ^{19}F 470.48 MHz; ^{31}P 202.41 MHz) or on a Bruker Avance III 500 HD spectrometer (^{1}H 500.20 MHz; ^{13}C 125.78 MHz; ^{19}F 470.66 MHz; ^{31}P 202.48 MHz). Positive shifts are downfield from the external standards TMS (^{1}H, ^{13}C), CCl_3F (^{19}F) and H_3PO_4 (^{31}P). The NMR spectra were recorded in the indicated deuterated solvent or in relation to acetone-d_6-filled capillaries.

1.2.2 IR Spectroscopy

IR spectra were recorded on an ALPHA-FT-IR spectrometer (Bruker) using an ATR unit with a diamond crystal for liquids and solids.

1.2.3 Elemental Analyses

Elemental analyses were performed by Mikroanalytisches Laboratorium Kolbe (Oberhausen, Germany). The elemental analysis of **[2H][$^{\mathbf{MeO}t\mathbf{Bu2}}$PhO]** was performed in the element-analytical laboratory of the Universität Bielefeld using the EURO EA Element Analyzer 2010 (HEKAtech GmbH).

1.2.4 Melting Point

Melting points were measured on a Mettler Toledo Mp70 Melting Point System.

1.2.5 Cyclic Voltammetry

The cyclic voltammetric investigations were performed on a PGSTAT101 potentiostat (Metrohm) using a „three-electrode arrangement“ in a flame-dried 25 mL Schlenk flask under inert atmosphere with a glassy carbon working electrode (2.0(1) mm diameter), a counter electrode (stainless steel 18/8, 2.0(1) mm diameter) and an Ag/AgCl reference electrode in a saturated ethanolic LiCl solution (148 mV vs. SHE). The supporting electrolyte $[NBu_4][PF_6]$ was carefully dried in a high vacuum (10^{-3} mbar). THF was dried over K and freshly distilled prior to use. For every run 0.1 mmol of the substrate and 15 mL of the electrolyte solution were used. The Fc/Fc^+ couple was used as internal standard by adding a small amount (spatula tip) of ferrocene after the measurements. The obtained redox potentials were finally recalculated based on the Fc/Fc^+ couple which was set at +0.405 V vs. SCE.

1.2.6 Mass spectrometry

Nano-ESI mass spectra were recorded using an Esquire 3000 ion trap mass spectrometer (Bruker Daltonik GmbH, Bremen, Germany) equipped with a nano-ESI source. Samples were dissolved in THF and introduced to static nano-ESI using *in-house* pulled glass emitters. Nitrogen served both as nebulizer gas and dry gas. Nitrogen was generated by a Bruker nitrogen generator NGM 11. Helium served as cooling gas for the ion trap and collision gas for mass spectrometry experiments. The mass axis was externally calibrated with ESI-L Tuning Mix (Agilent Technologies, Santa Clara, CA, USA) as calibration standard.

1.3 Syntheses

1.3.1 Synthesis of [1H][PhO(HOPh)]

Phosphazene **1** (524 mg, 1.68 mmol) is dissolved in diethyl ether (10 mL) and phenol (332 mg, 3.528 mmol) is added at ambient temperature. The resulting two-phase system is stirred for 15 minutes and then cooled at -28 °C overnight. The supernatant is removed via a syringe and the product (725 mg, 1.45 mmol, 86 % based on **1**) is isolated as a slight brown crystalline solid after drying in a high vacuum (m.p. > 63 °C).

^{1}H NMR (THF-d$_8$, rt): δ [ppm] = 1.3 (s, 9 H, C(CH$_3$)$_3$), 1.8 (m, 12 H, NCH$_2$C**H**$_2$), 3.2 (t, d, $^{3}J_{HH}$ = 7 Hz, $^{3}J_{PH}$ = 4 Hz, 12 H, NCH$_2$), 6.4 (m, 2 H, *para* H), 6.8 (m, 4 H, *ortho* H), 7.0 (m, 4 H, *meta* H), 11.1 (s, OH).
^{13}C NMR (THF-d$_8$, rt): δ [ppm] = 26.0 (d, $^{3}J_{PC}$ = 8 Hz, NCH$_2$**C**H$_2$), 31.8 (d, $^{3}J_{PC}$ = 5 Hz, C(**C**H$_3$)$_3$), 47.3 (d, $^{3}J_{PC}$ = 5 Hz, N**C**H$_2$CH$_2$), 52.2 (d, $^{2}J_{PC}$ = 1 Hz, **C**(CH$_3$)$_3$), 114.4 (s, *para* C), 117.0 (s, *ortho* C), 128.3 (s, *meta* C), 163.2 (s, *ipso* C).
^{31}P NMR (THF-d$_8$, rt): δ [ppm] = 16.3 (s).
IR (ATR): $\tilde{\nu}$ [cm^{-1}] = 3209 (vw, br), 3049 (vw), 2970 (w), 2872 (w), 2691 (w), 2559 (w, br), 1895 (vw), 1807 (vw), 1704 (vw), 1583 (w), 1468 (m), 1412 (w), 1392 (w), 1367 (w), 1347 (w), 1294 (w), 1243 (s), 1226 (s), 1199 (s), 1161 (m), 1128 (m), 1077 (vs), 1017 (vs), 984 (s), 916 (m), 866 (m), 837 (m), 817 (m), 752 (vs), 692 (vs), 617 (m), 582 (m), 567 (m), 547 (m), 514 (s), 493 (s), 453 (m), 435 (m), 403 (m).
elemental analysis of $C_{28}H_{45}N_4O_2P$ (M = 500.7 g/mol): calcd.: C 67.17, H 9.06, N 11.19; found: C 67.07, H 9.14, N 11.13.

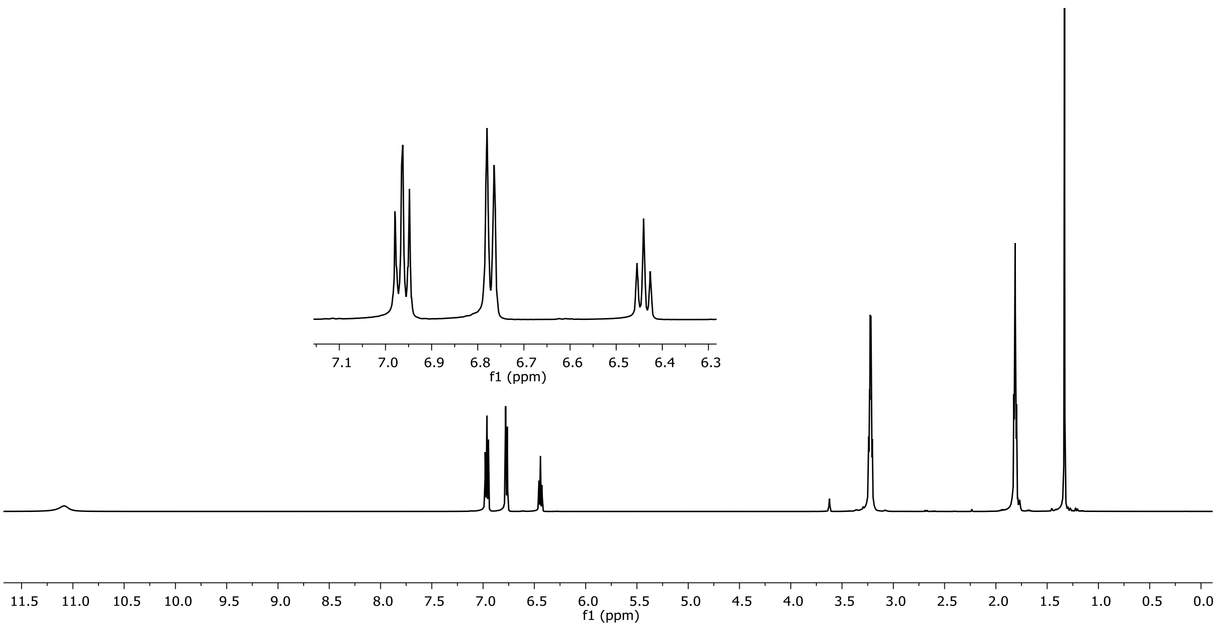

Figure 1. ^{1}H NMR spectrum of **[1H][PhO(HOPh)]** in THF-d$_8$ (500 MHz).

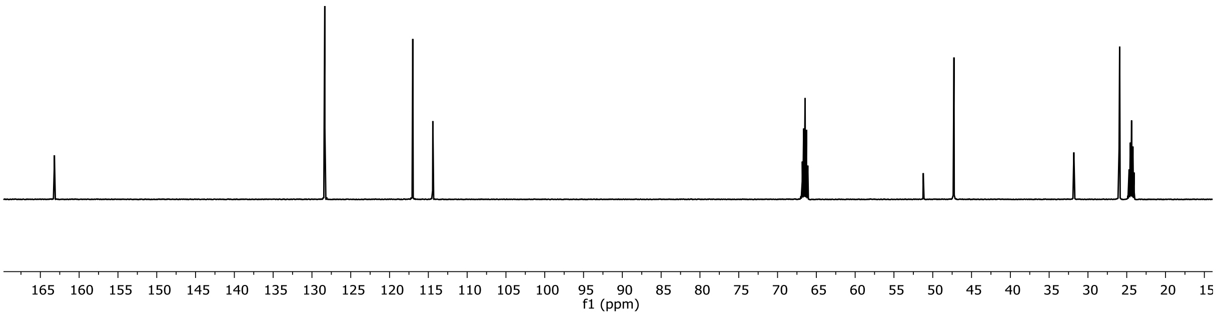

Figure 2. ^{13}C NMR spectrum of **[1H][PhO(HOPh)]** in THF-d$_8$ (500 MHz).

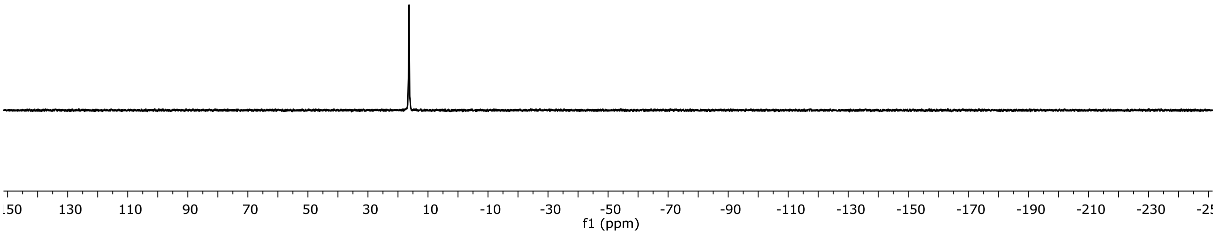

Figure 3. ^{31}P NMR spectrum of **[1H][PhO(HOPh)]** in THF-d$_8$ (500 MHz).

1.3.2 Synthesis of [2H][PhO]

Phosphazene **2** (1.40 g, 1.57 mmol) is dissolved in diethyl ether (7 mL) and phenol (149 mg, 1.58 mmol) is rapidly added in 3 mL of diethyl ether. The emulsion is stirred for 1.5 hours and cooled to -28 °C overnight, by which a colorless solid precipitates. For the complete precipitation of the product *n*-hexane (4 mL) is added and the supernatant is removed via a syringe. The solid is washed with 4 mL of *n*-hexane and dried in a high vacuum. The product (1.46 g, 1.49 mmol, 95 % based on **2**) is isolated as a colorless solid (dec. > 75 °C).

^{1}H NMR (THF-d_8, rt): δ [ppm] = 1.2 (t, $^{3}J_{HH}$ = 7 Hz, 54 H, CH_3), 1.4 (s, 9 H, $C(CH_3)_3$), 2.3 (d, $^{2}J_{PH}$ = 8 Hz, 1 H, NH), 3.2 (d, q, $^{3}J_{PH}$ = 10 Hz, $^{3}J_{HH}$ = 7 Hz, 36 H, CH_2), 5.5 (s, 1 H, *para* H), 6.0 (m, 2 H, *ortho* H), 6.6 (m, 2 H, *meta* H).
^{13}C NMR (THF-d_8, rt): δ [ppm] = 13.0 (d, $^{3}J_{PC}$ = 4 Hz, CH_3), 31.2 (d, $^{3}J_{PC}$ = 5 Hz, $C(\mathbf{C}H_3)_3$), 39.2 (d, $^{2}J_{PC}$ = 6 Hz, CH_2), 50.5 (d, $^{2}J_{PC}$ = 4 Hz, $\mathbf{C}(CH_3)_3$), 101.8 (s, *para* C), 119.4 (s, *ortho* C), 127.6 (s, *meta* C), 175.0 (s, *ipso* C).
^{31}P NMR (THF-d_8, rt): δ [ppm] = -33.5 (q, d, $^{2}J_{PP}$ = 70 Hz, $^{2}J_{PH}$ = 7 Hz, 1 P, P=NH), 7.8 (d, tridec, $^{2}J_{PP}$ = 70 Hz, $^{3}J_{PH}$ = 10 Hz, 3 P, $(Et_2N)_3P$).
IR (ATR): $\tilde{\nu}$ [cm^{-1}] = 2969 (w), 2930 (w), 2870 (w), 1578 (w), 1538 (vw), 1485 (w), 1462 (w), 1414 (w), 1377 (m), 1350 (w), 1330 (w), 1261 (s), 1227 (m), 1201 (s), 1174 (vs), 1108 (w), 1076 (w), 1054 (w), 1017 (vs), 979 (w), 941 (vs), 848 (m), 795 (s), 740 (m), 699 (s), 691 (s), 614 (m), 590 (w), 509 (vs), 456 (s), 435 (s).
elemental analysis of $C_{46}H_{106}N_{13}OP_4$ (M = 981.3 g/mol): calcd.: C 56.36, H 10.80, N 18.57; found: C 55.66, H 10.69, N 18.31.

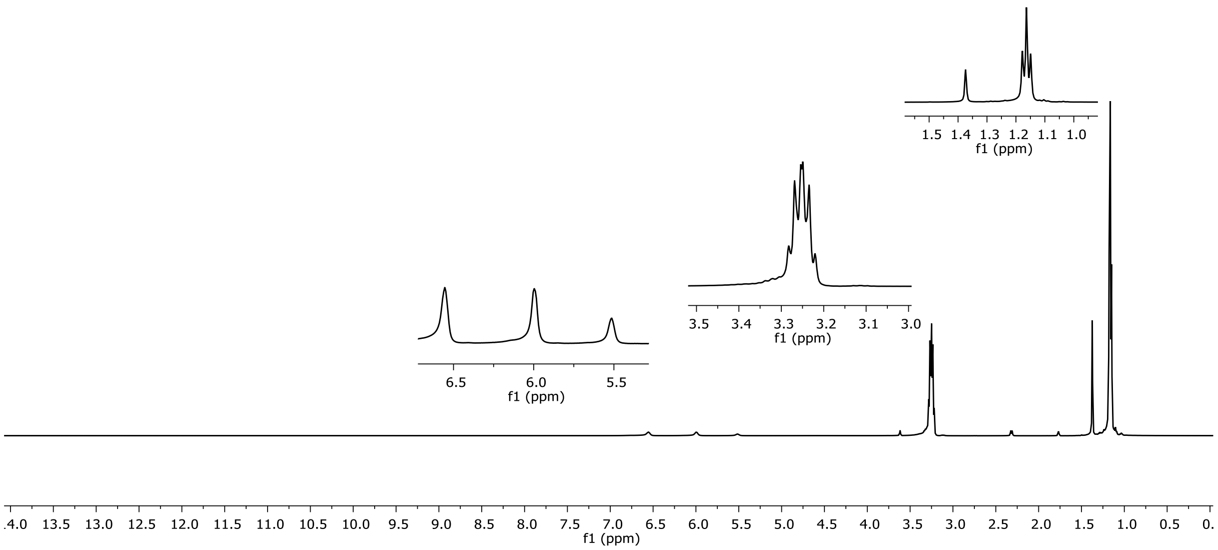

Figure 4. ^{1}H NMR spectrum of **[2H][PhO]** in THF-d_8 (500 MHz).

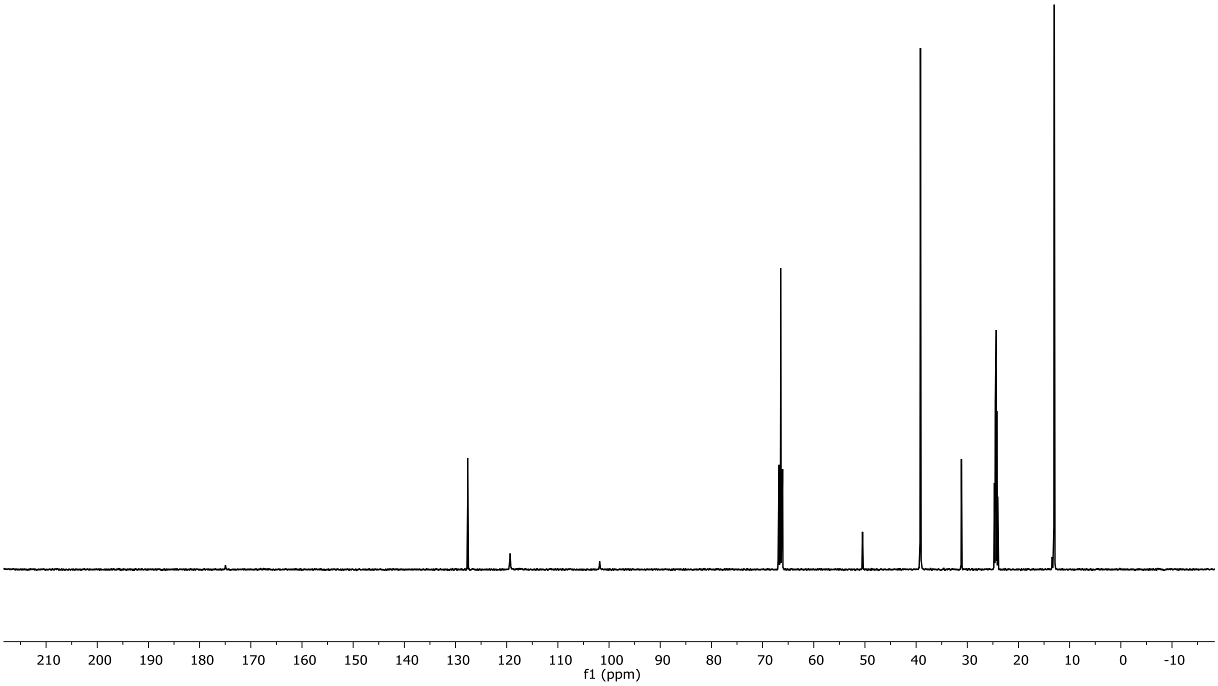

Figure 5. ^{13}C NMR spectrum of **[2H][PhO]** in THF-d_8 (500 MHz).

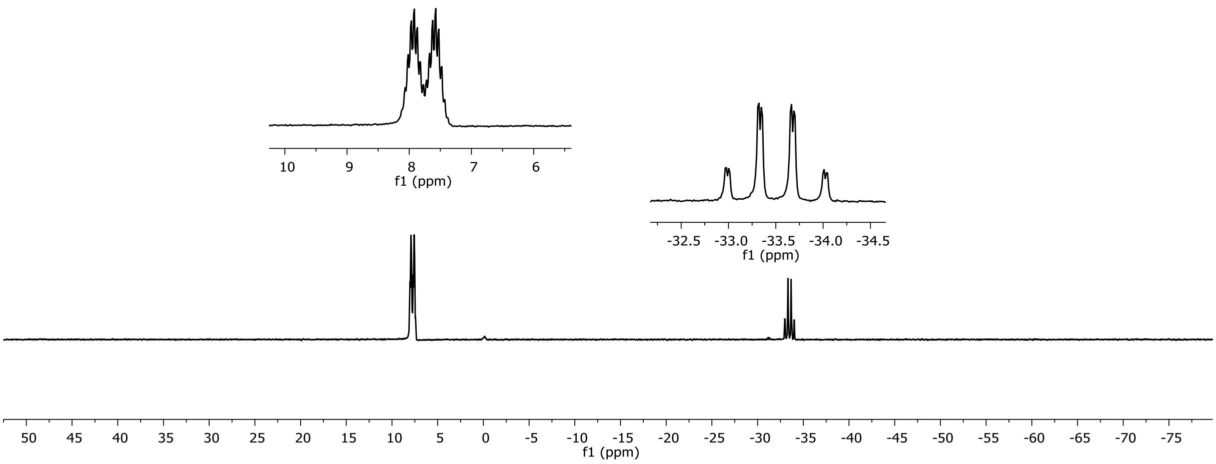

Figure 6. ^{31}P NMR spectrum of **[2H][PhO]** in THF-d_8 (500 MHz).

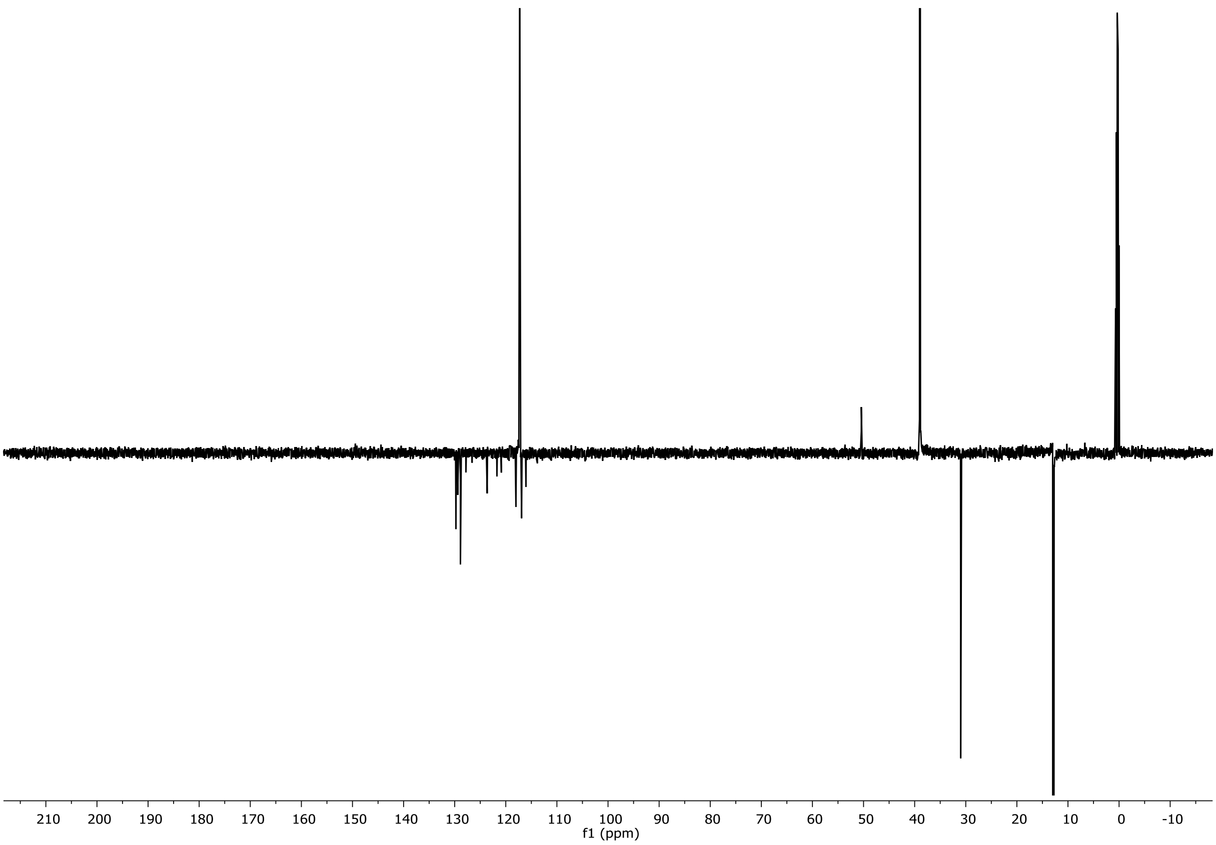

Figure 7. ^{13}C{^{1}H} APT NMR spectrum of the resulting decomposition products of **[2H][PhO]** in acetonitrile-d_3 (yellow solution) (500 MHz).

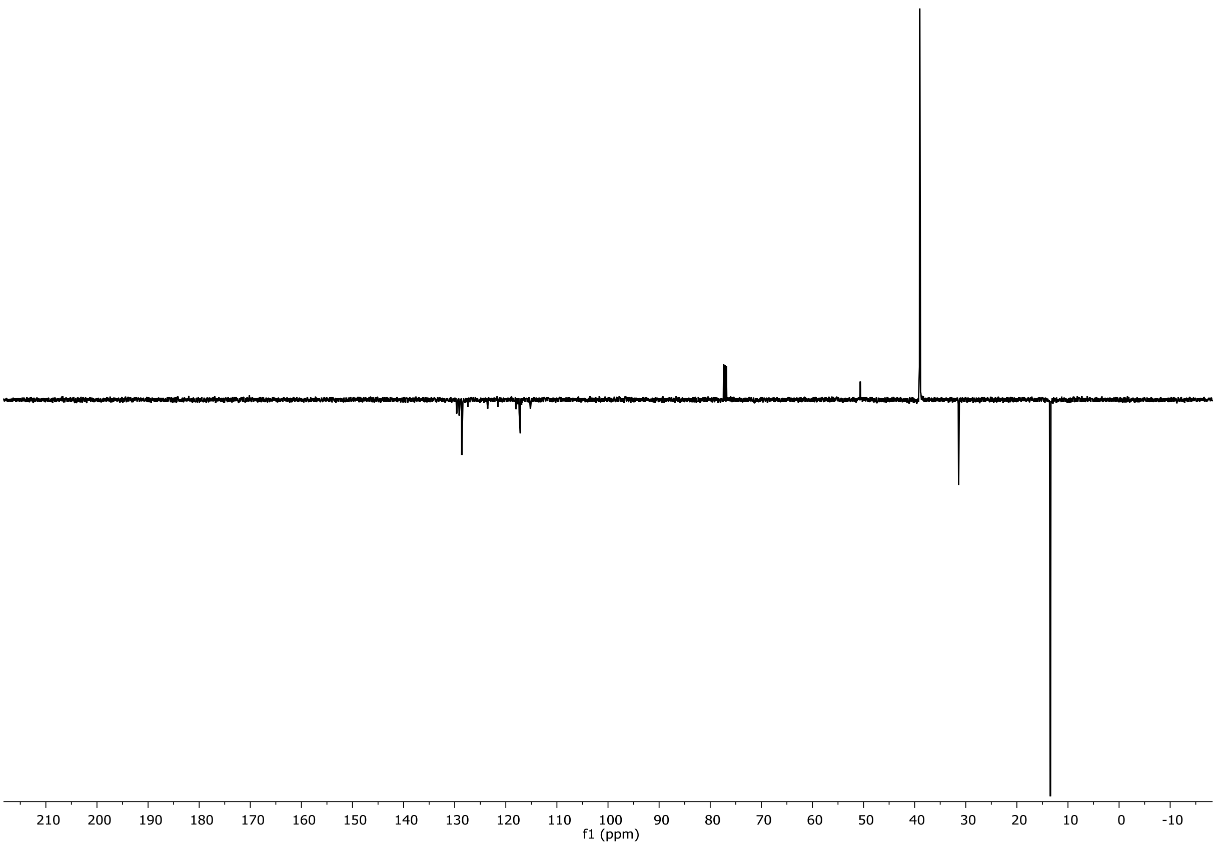

Figure 8. $^{13}C\{^{1}H\}$ APT NMR spectrum of the resulting decomposition products of **[2H][PhO]** in chloroform-d_1 (blue solution) (500 MHz).

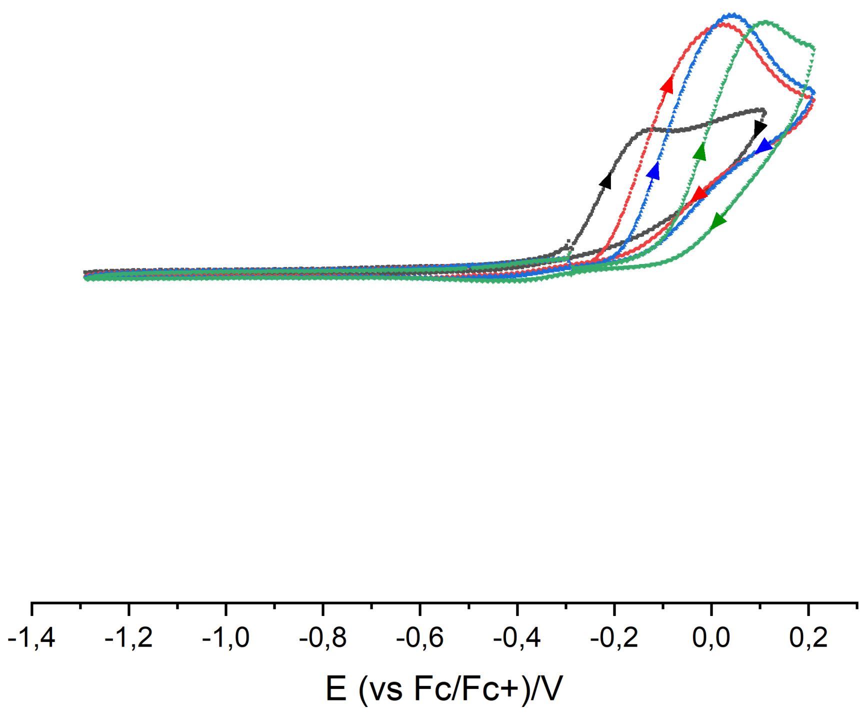

Figure 9. Cyclovoltammograms of **[2H][PhO]** (black) and **[2H][PhO]** with addition of H_2O to obtain concentrations of 0.1 M (28 mg H_2O, red), 0.2 M (59 mg H_2O, blue) and 0.7 M (258 mg H_2O, green) water in the electrolyte solution. Voltammograms recorded in 0.1 M $[NBu_4][PF_6]$ THF solution at 100 mV/s under inert atmosphere with a glassy carbon working electrode (2.0(1) mm), a counter electrode (steel 18/8, 2.0(1) mm) and an Ag/AgCl reference electrode. All potentials were recalculated using the Fc/Fc^+ couple (+0.405 V vs SCE).

1.3.3 Synthesis of [2H][$^{MeO\textit{t}Bu2}$PhO]

Phosphazene **2** (4.41 g, 4.98 mmol) is dissolved in diethyl ether (15 mL) and 2,6-di-*tert*-butyl-4-methoxyphenol ($^{MeO\textit{t}Bu2}$PhOH, 1.18 g, 4.99 mmol) in 5 mL diethyl ether is rapidly added to yield an intensely yellow suspension. After stirring for one hour *n*-hexane (10 mL) is added and the supernatant is removed via a syringe. The solid is washed with *n*-hexane (10 mL) and dried in a high vacuum. The product (5.46 g, 4.86 mmol, 98 % based on **2**) is isolated as a highly air sensitive yellow crystalline solid.

The product rapidly decomposes in a melting point capillary upon heating over 40 °C to yield a dark brown solid.

The product dissolves rapidly in acetonitrile and chloroform to yield intensely orange or light pink solutions.

^{1}H NMR (THF-d$_8$, rt): δ [ppm] = 1.2 (t, $^{3}J_{HH}$ = 7 Hz, 54 H, NCH$_2$C**H**$_3$), 1.4 (s, 9 H, NC(CH$_3$)$_3$), 1.4 (s, 18 H, C(CH$_3$)$_3$), 2.2 (d, $^{2}J_{PH}$ = 8 Hz, 1 H, NH), 3.2 (d, q, $^{3}J_{PH}$ = 10 Hz, $^{3}J_{HH}$ = 7 Hz, 36 H, NC**H**$_2$CH$_3$), 3.5 (s, 3 H, OCH$_3$), 6.4 (s, 2 H, *meta* H).

^{13}C NMR (THF-d$_8$, rt): δ [ppm] = 13.0 (d, $^{3}J_{PC}$ = 4 Hz, NCH$_2$**C**H$_3$), 29.6 (s, C(**C**H$_3$)$_3$), 31.1 (d, $^{3}J_{PC}$ = 5 Hz, NC(**C**H$_3$)$_3$), 35.0 (s, **C**(CH$_3$)$_3$), 39.1 (d, $^{2}J_{PC}$ = 6 Hz, N**C**H$_2$CH$_3$), 50.5 (d, $^{2}J_{PC}$ = 4 Hz, N**C**(CH$_3$)$_3$), 57.6 (s, OCH$_3$), 110.8 (s, *meta* C), 133.3 (s, *ortho* C), 140.7 (s, *para* C), 168.0 (s, *ipso* C).

^{31}P NMR (THF-d$_8$, rt): δ [ppm] = -33.7 (q, d, $^{2}J_{PP}$ = 70 Hz, $^{2}J_{PH}$ = 7 Hz, 1 P, P=NH), 7.7 (d, tridec, $^{2}J_{PP}$ = 70 Hz, $^{3}J_{PH}$ = 10 Hz, 3 P, (Et$_2$N)$_3$P).

IR (ATR): $\tilde{\nu}$ [cm^{-1}] = 2966 (vw), 2931 (vw), 2870 (w), 1465 (w), 1415 (w), 1377 (w), 1348 (w), 1244 (m), 1201 (s), 1173 (vs), 1102 (w), 1073 (vw), 1053 (w), 1016 (vs), 944 (s), 921 (m), 890 (w), 844 (w), 783 (s), 740 (w), 700 (s), 616 (w), 508 (s), 440 (m).

MS (ESI, pos.) {*m/z* (%) [assignment]}: 887.7 (100) **[2H]$^{+}$**

MS (ESI, neg.) {*m/z* (%) [assignment]}: 235.1 (100) **[$^{MeO\textit{t}Bu2}$PhO]$^{-}$**

elemental analysis of $C_{55}H_{123}N_{13}O_2P_4$ (M = 1122.6 g/mol): calcd.: C 58.85, H 11.04, N 16.22; found: C 59.24, H 10.74, N 16.12.

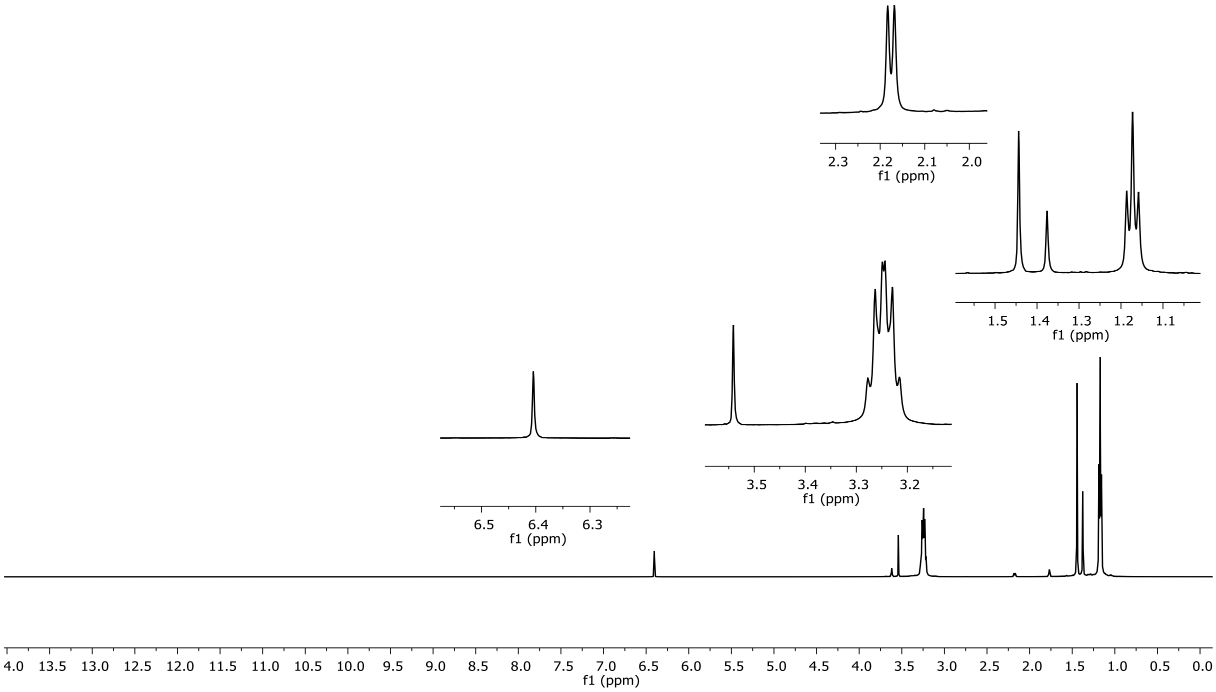

Figure 10. ^{1}H NMR spectrum of **[2H][$^{\mathrm{MeO}t\mathrm{Bu2}}$PhO]** in THF-$d_8$ (500 MHz).

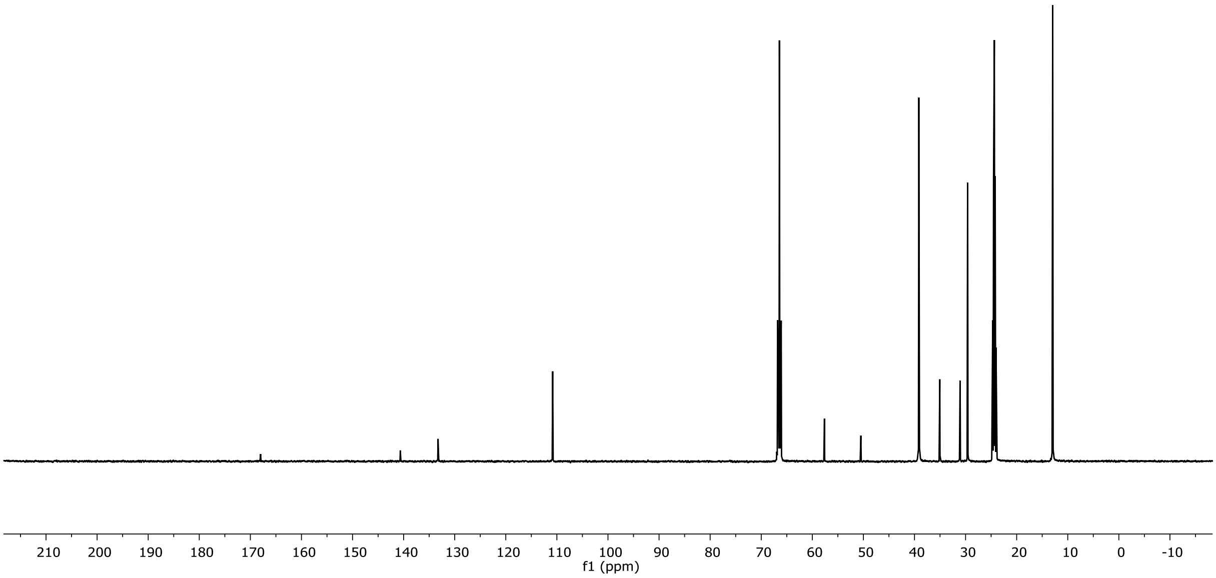

Figure 11. ^{13}C NMR spectrum of **[2H][$^{\mathrm{MeO}t\mathrm{Bu2}}$PhO]** in THF-$d_8$ (500 MHz).

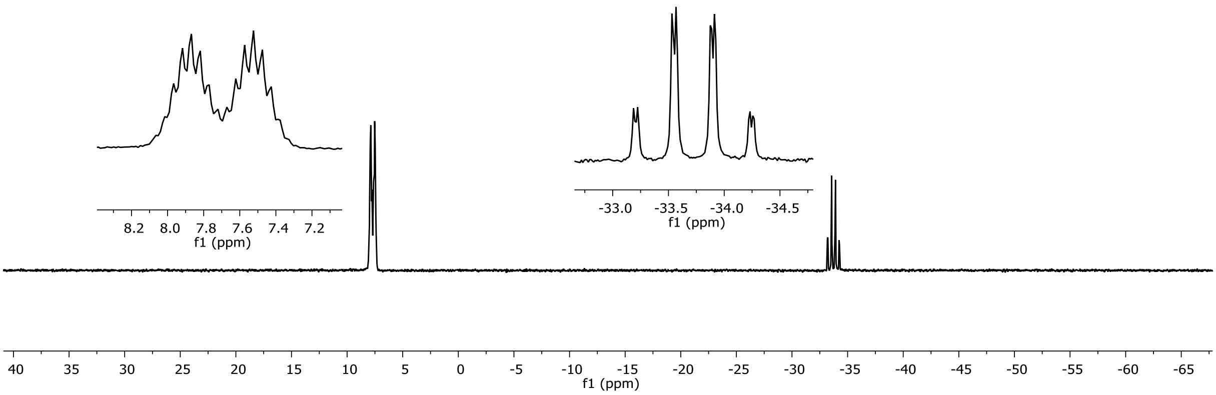

Figure 12. ^{31}P NMR spectrum of **[2H][^{MeOtBu2}PhO]** in THF-d$_8$ (500 MHz).

1.3.4 Synthesis of [2H][PhO(HOPh)]

Phosphazene **2** (2.25 g, 2.54 mmol) is dissolved in 15 mL of diethyl ether and phenol (0.48 g, 5.09 mmol) in 4 mL of diethyl ether is rapidly added. Immediately a second yellowish phase forms. The emulsion is stirred for an additional hour and then cooled to -28 °C overnight. The supernatant is removed via a syringe and the solid is dried in a high vacuum. The product (2.69 g, 2.50 mmol, 99 %, based on phenol) is isolated as a light-brown crystalline solid (m.p. > 125 °C (dec.)).

^{1}H NMR (THF-d$_8$, rt): δ [ppm] = 1.1 (t, $^{3}J_{HH}$ = 7 Hz, 54 H, NCH$_2$C**H**$_3$), 1.4 (s, 9 H, NC(CH$_3$)$_3$), 2.1 (d, $^{2}J_{PH}$ = 8 Hz, 1 H, NH), 3.2 (d, q, $^{3}J_{PH}$ = 10 Hz, $^{3}J_{HH}$ = 7 Hz, 36 H, NC**H**$_2$CH$_3$), 6.1 (m, 2 H, *para* H), 6.6 (m, 4 H, *ortho* H), 6.8 (m, 4 H, *meta* H), 15.1 (s, OH).
^{13}C NMR (THF-d$_8$, rt): δ [ppm] = 12.9 (d, $^{3}J_{PC}$ = 4 Hz, NCH$_2$**C**H$_3$), 31.1 (d, $^{3}J_{PC}$ = 5 Hz, NC(**C**H$_3$)$_3$), 39.1 (d, $^{2}J_{PC}$ = 6 Hz, N**C**H$_2$CH$_3$), 50.5 (d, $^{2}J_{PC}$ = 4 Hz, N**C**(CH$_3$)$_3$), 110.7 (s, *para* C), 117.4 (s, *ortho* C), 127.7 (s, *meta* C), 167.2 (s, *ipso* C).
^{31}P NMR (THF-d$_8$, rt): δ [ppm] = -33.7 (q, d, $^{2}J_{PP}$ = 70 Hz, $^{2}J_{PH}$ = 8 Hz, 1 P, P=NH), 7.7 (d, tridec, $^{2}J_{PP}$ = 70 Hz, $^{3}J_{PH}$ = 10 Hz, 3 P, (Et$_2$N)$_3$P).
IR (ATR): $\tilde{\nu}$ [cm^{-1}] = 2973 (vw), 2965 (w), 2949 (vw), 2934 (vw), 2867 (vw), 1455 (vw), 1376 (w), 1353 (w), 1260 (s), 1222 (w), 1201 (m), 1173 (s), 1157 (s), 1103 (w), 1074 (w), 1056 (w), 1020 (vs), 943 (vs), 867 (w), 845 (w), 822 (m), 782 (s), 750 (s), 695 (vs), 620 (m), 603 (m), 541 (s), 511 (vs), 494 (vs), 482 (m), 445 (s), 408 (vs).
elemental analysis of $C_{52}H_{111}N_{13}O_2P_4$ (M = 1074.4 g/mol): calcd.: C 58.13, H 10.41, N 16.95; found: C 57.89, H 10.22, N 17.03.

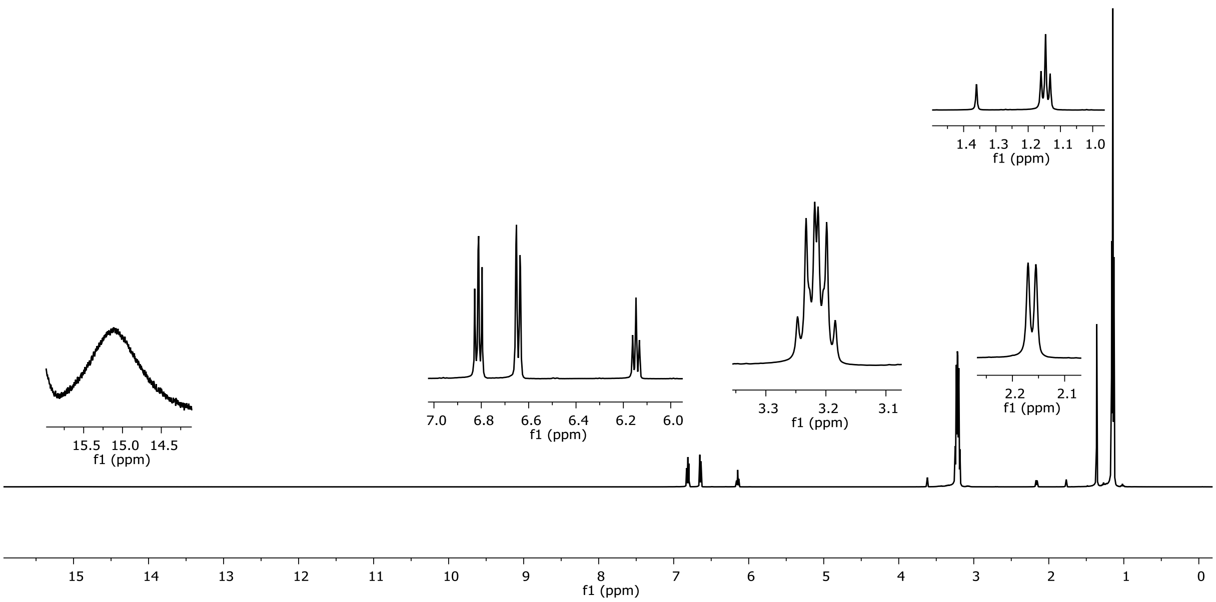

Figure 13. ^{1}H NMR spectrum of **[2H][PhO(HOPh)]** in THF-d8 (500 MHz).

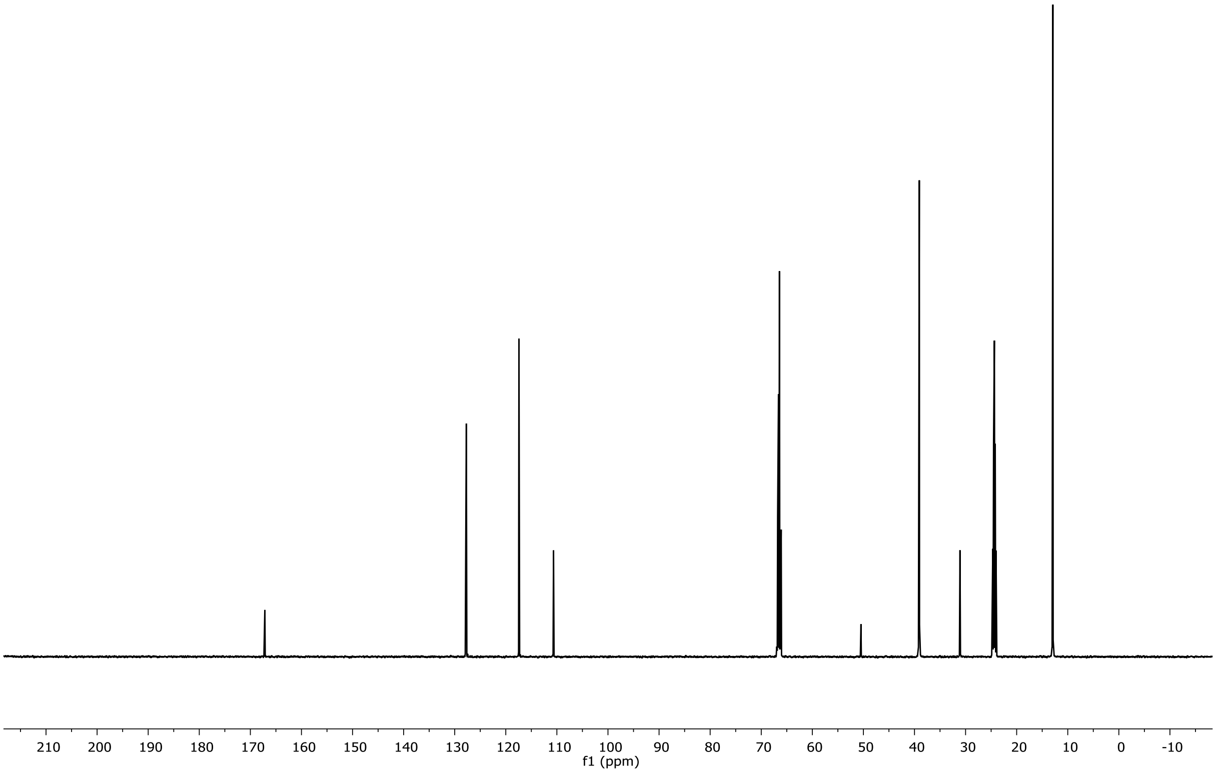

Figure 14. ^{13}C NMR spectrum of **[2H][PhO(HOPh)]** in THF-d8 (500 MHz).

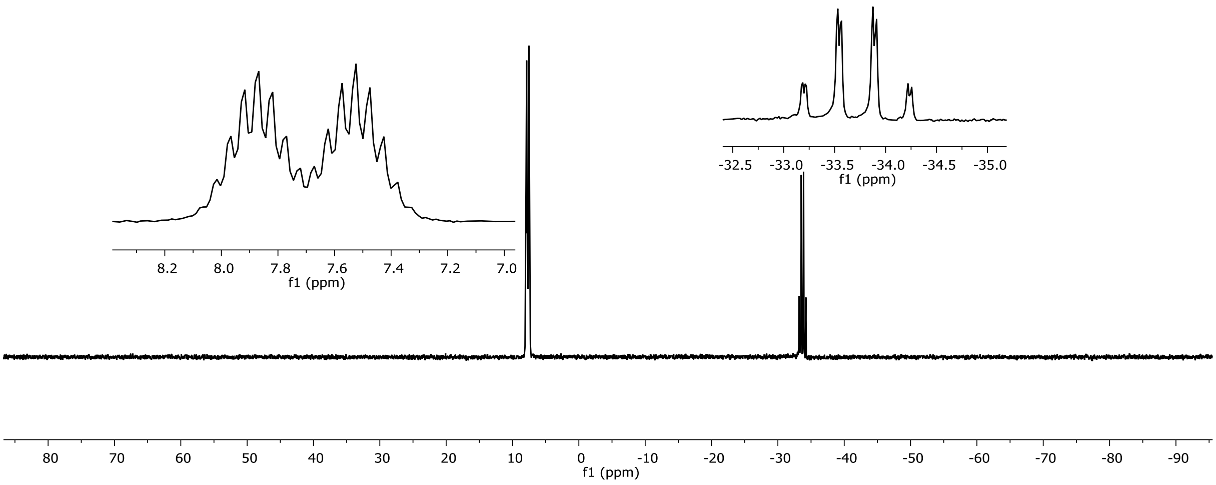

Figure 15. ^{31}P NMR spectrum of **[2H][PhO(HOPh)]** in THF-d_8 (500 MHz).

1.3.5 Activation of SF_6

At -196 °C diethyl ether (15 mL) is condensed onto the salt **[2H][^{MeOtBu2}PhO]** (514 mg, 0.46 mmol), the resulting yellowish emulsion is shortly allowed to warm to room temperature and then sulfur hexafluoride (SF_6) (2.35 mmol) is condensed onto the mixture at -196 °C. The emulsion is again warmed to room temperature and stirred for 4 days. The deep red supernatant of the resulting suspension is removed via a syringe at -40 °C and the solid is washed with cold diethyl ether (3 x 5 mL). A salt mixture of **[2H][SF_5]** and **[2H][F]** (389 mg, 0.38 mmol, 88 %) is isolated as a colorless, highly hygroscopic crystalline solid (dec. > 123 °C).

^{13}C NMR (THF, rt): δ [ppm] = 13.2 (d, $^3J_{PC}$ = 4 Hz, $NCH_2\mathbf{C}H_3$), 31.4 (d, $^3J_{PC}$ = 5 Hz, $NC(\mathbf{C}H_3)_3$), 39.4 (d, $^2J_{PC}$ = 6 Hz, $N\mathbf{C}H_2CH_3$), 50.8 (d, $^2J_{PC}$ = 4 Hz, $N\mathbf{C}(CH_3)_3$).
^{19}F NMR (THF, rt): δ [ppm] = -173.0 (s, br, F^-), 59.4 (s, br, 4 F, $[SF_5]^-$), 88.9 (s, br, 1 F, $[SF_5]^-$).
^{31}P NMR (THF, rt): δ [ppm] = -33.3 (q, d, $^2J_{PP}$ = 70 Hz, $^2J_{PH}$ = 8 Hz, 1 P, P=NH), 8.1 (d, tridec, $^2J_{PP}$ = 70 Hz, $^3J_{PH}$ = 10 Hz, 3 P, $(Et_2N)_3P$).
IR (ATR): $\tilde{\nu}$ [cm^{-1}] = 2966 (vw), 2928 (vw), 2869 (vw), 1774 (br, vw), 1463 (vw), 1414 (vw), 1377 (w), 1349 (w), 1259 (m), 1227 (w), 1202 (m), 1173 (s), 1107 (w), 1054 (w), 1018 (vs), 942 (s), 846 (w), 785 (m), 741 (w), 700 (s), 613 (w), 579 (vs), 511 (vs), 458 (vs), 437 (vs).

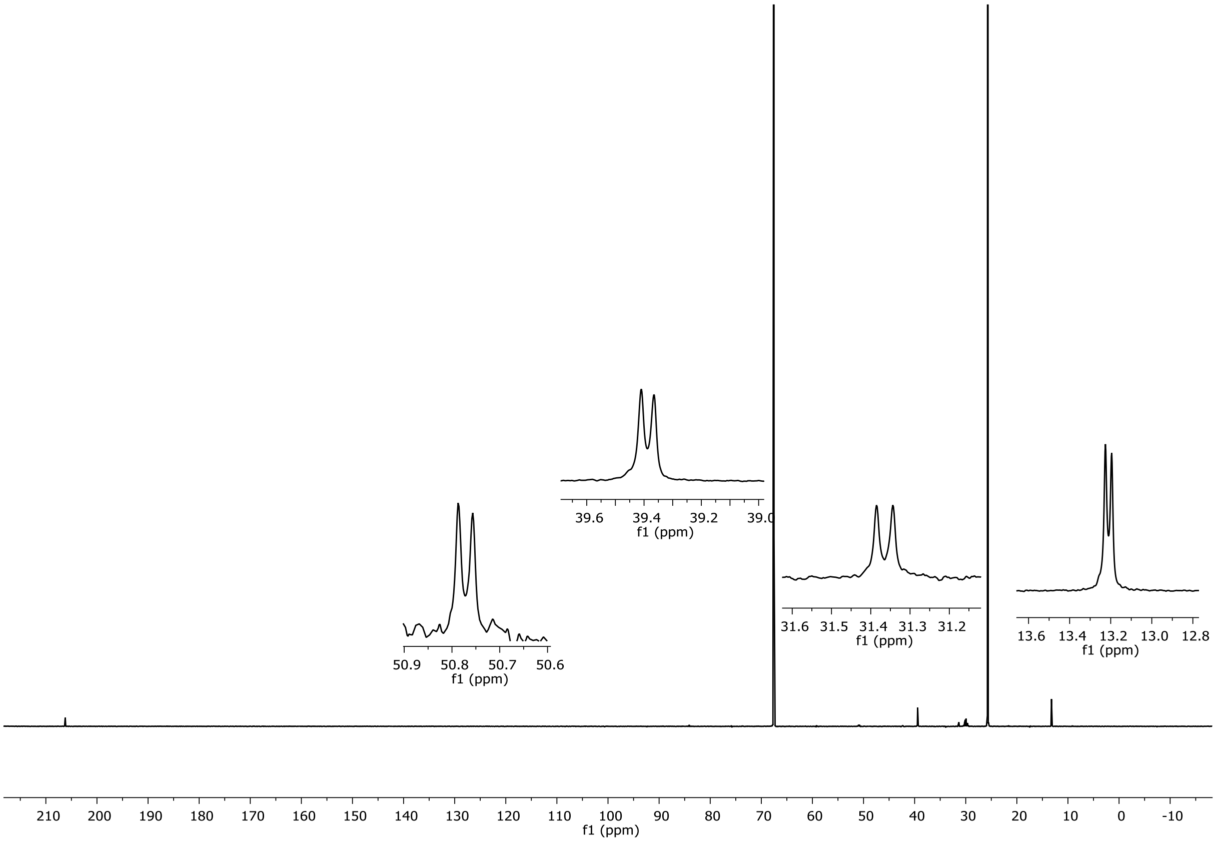

Figure 16. $^{13}C\{^{1}H\}$ NMR spectrum of the salt mixture **[2H][SF$_5$]** and **[2H][F]** in THF (500 MHz). Lock with acetone-d$_6$ in a capillary.

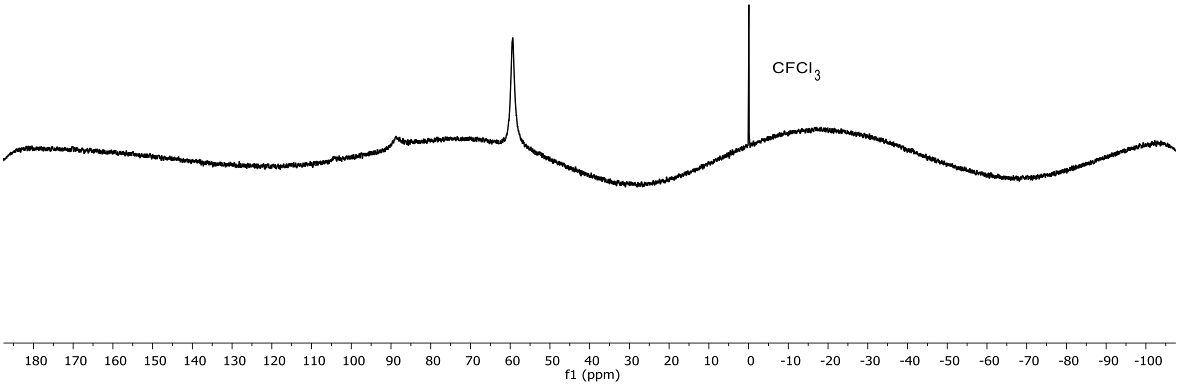

Figure 17. ^{19}F NMR spectrum of the salt mixture **[2H][SF$_5$]** and **[2H][F]** in THF (500 MHz). Lock with acetone-d$_6$ in a capillary. $CFCl_3$ as internal standard. SW [ppm] 295.08; O1P [ppm] 40.00.

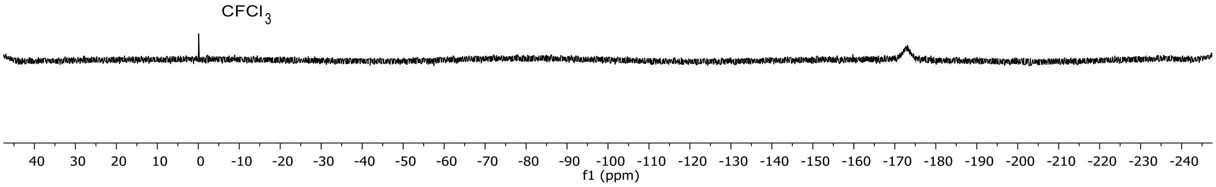

Figure 18. ^{19}F NMR spectrum of the salt mixture **[2H][SF₅]** and **[2H][F]** in THF (500 MHz). Lock with acetone-d₆ in a capillary. $CFCl_3$ as internal standard. SW [ppm] 295.12; O1P [ppm] -100.00.

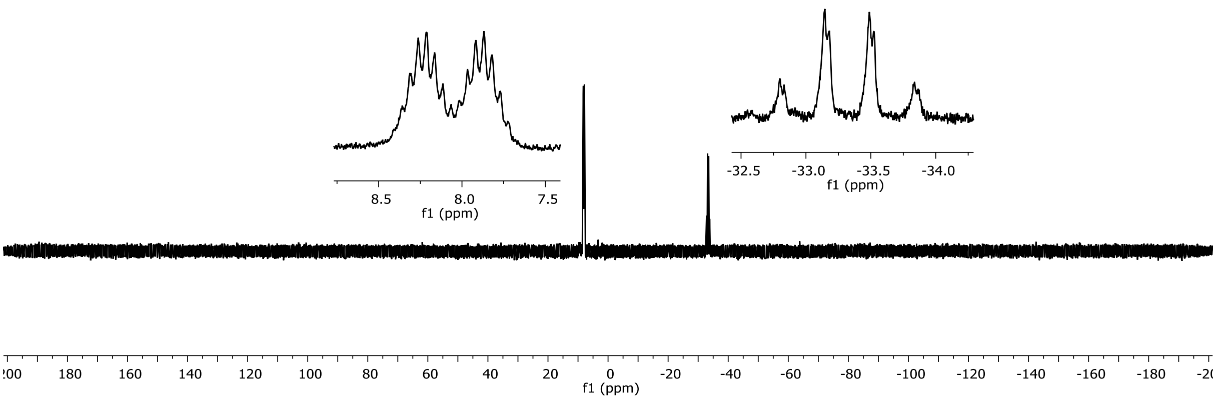

Figure 19. ^{31}P NMR spectrum of the salt mixture **[2H][SF₅]** and **[2H][F]** in THF (500 MHz). Lock with acetone-d₆ in a capillary.

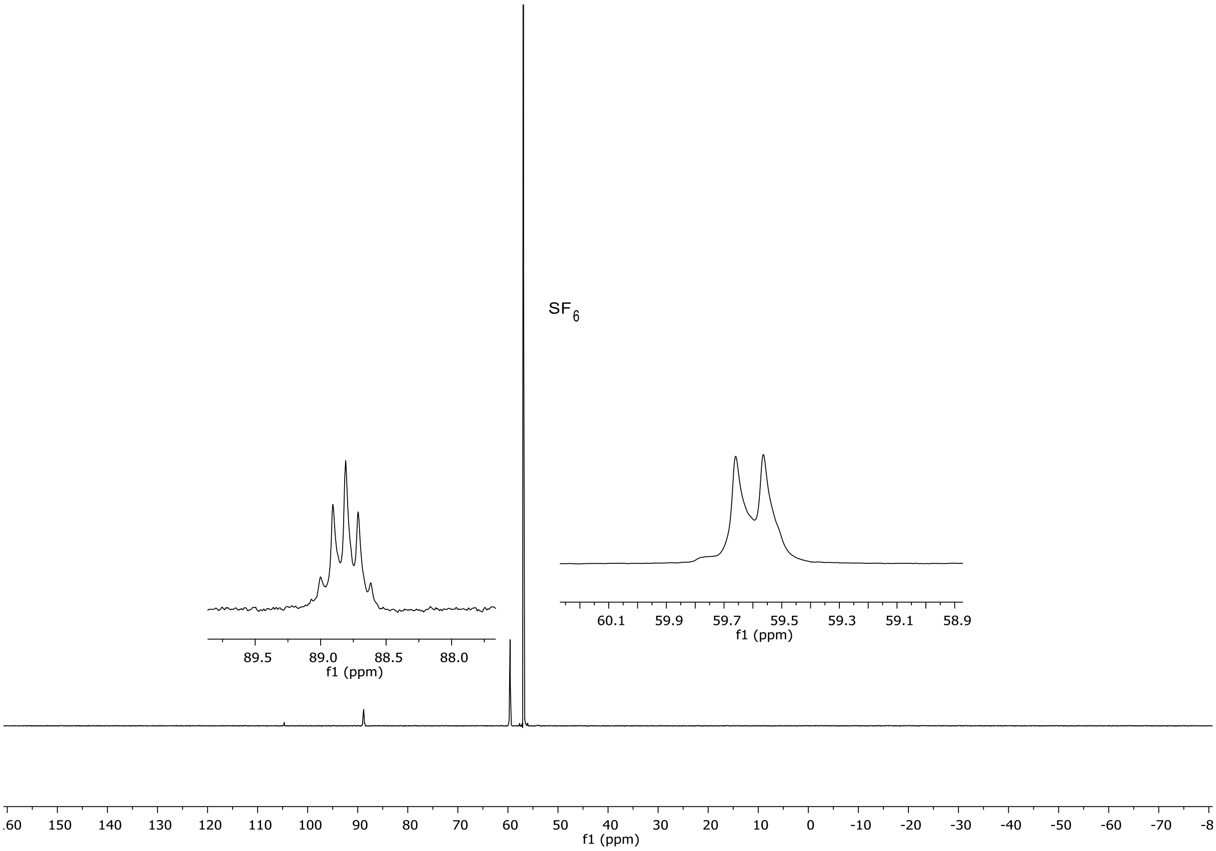

Figure 20. ^{19}F NMR spectrum of the SF_6 activation in Et_2O solution in an FEP tube (500 MHz). Lock with acetone-d_6 in a surrounding NMR tube. SW [ppm] 241.52; O1P [ppm] 40.00.

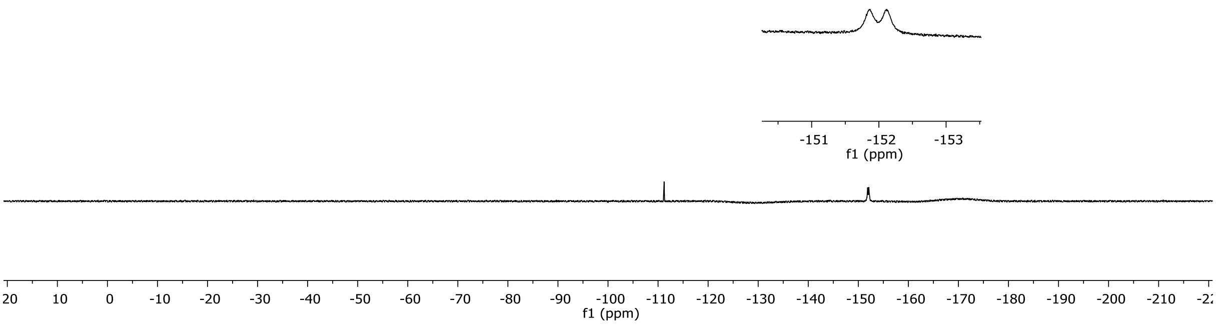

Figure 21. ^{19}F NMR spectrum of the SF_6 activation in Et_2O solution in an FEP tube (500 MHz). Lock with acetone-d_6 in a surrounding NMR tube. The resonance at δ = -152 ppm results from $[HF_2]^-$, which forms over time. SW [ppm] 241.56; O1P [ppm] -100.00.

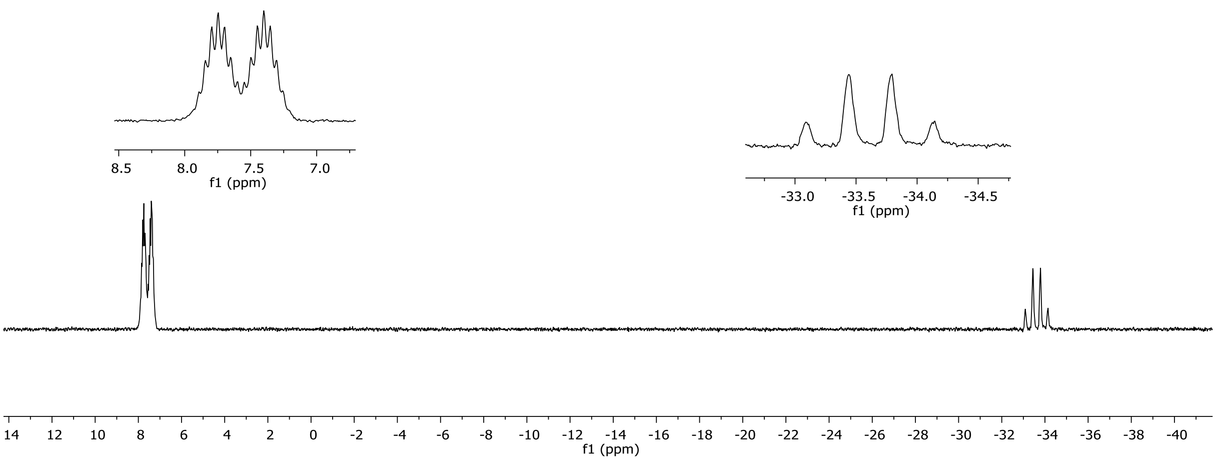

Figure 22. ^{31}P NMR spectrum of the SF_6 activation in Et_2O solution in an FEP tube (500 MHz). Lock with acetone-d_6 in a surrounding NMR tube.

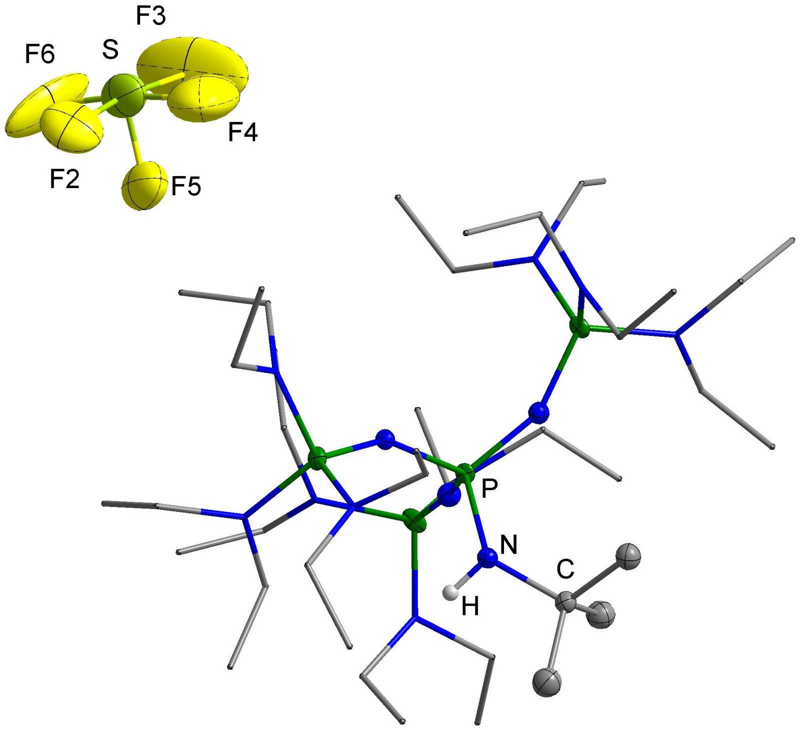

Figure 23. Molecular structure of **[2H][SF5]** in the solid state. Thermal ellipsoids are shown at 50 % probability. The hydrogen atoms bonded at carbon atoms are omitted for clarity. Diethylamino groups are shown as stick model.

1.4 Details on the X-Ray Diffraction

The crystal data were collected on a Rigaku Supernova diffractometer (Cu-K_α radiation (λ = 154.184 pm) or Mo-K_α radiation (λ = 71.073 pm) at 100.0(2) K.

Using Olex2[1], the structures were solved with the ShelXS[2] structure solution program using direct methods and refined with the ShelXL[3] refinement package using least squares minimization. All hydrogen atoms bonded at nitrogen or oxygen were refined isotropically including the 1:1 disordered ones in **[2H][PhO(HOPh)]**.

Details of the X-ray investigation are given in Tables 1-3. CCDC 1973242-1973244, 2002668 and 2002669 contain the supplementary crystallographic data for this paper. These data can be obtained free of charge via http://www.ccdc.cam.ac.uk/conts/retrieving.html.

Table 1. Structure refinement data of **[1H][PhO(HOPh)]** and **[2H][PhO]**.

compound	**[1H][PhO(HOPh)]**	**[2H][PhO]**
empirical formula	$C_{28}H_{45}N_4O_2P$	$C_{46}H_{105}N_{13}OP_4$
a / pm	1927.213(11)	1293.050(10)
b / pm	1640.156(10)	1952.430(10)
c / pm	8711.71(5)	2285.320(10)
α / °	90	90
β / °	90	102.3290(10)
γ / °	90	90
V / 10^6 pm^3	2753.71(3)	5636.44(6)
Z	4	4
ρ_{calc} / mg·mm^{-3}	1.208	1.155
crystal system	orthorhombic	monoclinic
space group	$Pna2_1$	$P2_1/n$
color shape	yellowish block	colorless irregular
crystal size / mm^{-3}	0.087 × 0.07 × 0.033	0.292 × 0.202 × 0.148
μ / mm^{-1}	1.122	1.581
F(000)	1088.0	2160.0
2θ range for data col. / °	7.078 to 151.786°	6.014 to 152.968°
index ranges	-24 ≤ *h* ≤ 24<br>-19 ≤ *k* ≤ 20<br>-10 ≤ *l* ≤ 10	-11 ≤ *h* ≤ 16<br>-22 ≤ *k* ≤ 24<br>-28 ≤ *l* ≤ 27
reflections col.	50317	27180
independent refl.	5686	11608
R(int)	0.0343	0.0156
data/restraints/	5686/1/327	11608/0/623

parameter		
goodness-of-fit on F^2	1.046	1.036
R_1 / wR_2 [I>2σ(I)]	0.0249/0.0665	0.0341/0.0883
R_1 / wR_2 (all data)	0.0256/0.0671	0.0357/0.0897
$\Delta\rho_{max/min}$ / e Å^{-3}	0.25/-0.16	0.74/-0.57
CCDC number	1973242	1973243

Table 2. Structure refinement data of **[2H][PhO(HOPh)]** and **[2H][$^{\mathbf{MeO}t\mathbf{Bu2}}$PhO]**.

compound	**[2H][PhO(HOPh)]**	**[2H][$^{\mathbf{MeO}t\mathbf{Bu2}}$PhO]**
empirical formula	$C_{52}H_{111}N_{13}O_2P_4$	$C_{55}H_{123}N_{13}O_2P_4$
a / pm	1332.764(8)	1646.26(2)
b / pm	1919.982(10)	1956.90(2)
c / pm	2416.372(14)	2058.51(2)
α / °	90	90
β / °	90.7361(5)	90
γ / °	90	90
V / 10^6 pm^3	6182.70(6)	6631.62(13)
Z	4	4
ρ_{calc} / mg·mm^{-3}	1.154	1.124
crystal system	monoclinic	orthorhombic
space group	$P2_1/c$	$P2_12_12_1$
color shape	colorless block	clear yellow, irregular
crystal size / mm^{-3}	0.244 × 0.144 × 0.135	0.221 × 0.19 × 0.087
μ / mm^{-1}	1.496	1.412
F(000)	2360.0	2480.0
2θ range for data col. / °	5.88 to 153.038°	6.232 to 152.94°

index ranges	-16 ≤ h ≤ 16<br>-24 ≤ k ≤ 24<br>-30 ≤ l ≤ 30	-20 ≤ h ≤ 20<br>-24 ≤ k ≤ 24<br>-25 ≤ l ≤ 16
reflections col.	126690	59589
independent refl.	12952	13795
R(int)	0.0404	0.0634
data/restraints/ parameter	12952/0/1127	13795/0/699
goodness-of-fit on F^2	1.016	1.030
R_1 / wR_2 [I>2σ(I)]	0.0300/0.0794	0.0413/0.1061
R_1 / wR_2 (all data)	0.0337/0.0823	0.0464/0.1102
$\Delta\rho_{max/min}$ / e Å^{-3}	0.36/-0.35	0.43/-0.36
CCDC number	1973244	2002668

Table 3. Structure refinement data of **[2H][SF$_5$]**.

compound	**[2H][SF$_5$]**
empirical formula	$C_{40}H_{100}F_5N_{13}P_4S$
a / pm	1263.54(9)
b / pm	1772.96(9)
c / pm	2459.20(15)
α / °	90
β / °	99.856(6)
γ / °	90
V / 10^6 pm^3	5427.8(6)
Z	4
ρ_{calc} / mg·mm^{-3}	1.241

crystal system	monoclinic
space group	$P2_1/c$
color shape	colorless plate
crystal size / mm^{-3}	0.218 × 0.063 × 0.023
μ / mm^{-1}	2.129
F(000)	2208.0
2θ range for data col. / °	9.978 to 133.182°
index ranges	$-14 \leq h \leq 15$<br>$-21 \leq k \leq 21$<br>$-21 \leq l \leq 29$
reflections col.	24230
independent refl.	24230
R(int)	0.1877
data/restraints/<br>parameter	11517/318/569
goodness-of-fit on F^2	1.656
R_1 / wR_2 [I>2σ(I)]	0.1927/0.4507
R_1 / wR_2 (all data)	0.2679/0.4931
$\Delta\rho_{max/min}$ / e $Å^{-3}$	2.63/-1.70
CCDC number	2002669

References

[1] O. V. Dolomanov, L. J. Bourhis, R. J. Gildea, J. A. K. Howard, H. Puschmann, *J. Appl. Cryst.* **2009**, *42*, 339.

[2] G. M. Sheldrick, *Acta Cryst. A* **2015**, *71*, 3.

[3] G. M. Sheldrick, *Acta Cryst. C* **2015**, *71*, 3.
